# The Outcome of Breast Cancer Is Associated with National Human Development Index and Health System Attainment

**DOI:** 10.1371/journal.pone.0158951

**Published:** 2016-07-08

**Authors:** Kaimin Hu, Lixia Lou, Wei Tian, Tao Pan, Juan Ye, Suzhan Zhang

**Affiliations:** 1 Cancer Institute, the Second Affiliated Hospital of Zhejiang University, College of Medicine, Hangzhou, Zhejiang, China; 2 Department of Ophthalmology, the Second Affiliated Hospital of Zhejiang University, College of Medicine, Hangzhou, Zhejiang, China; West Virginia University, UNITED STATES

## Abstract

Breast cancer is a worldwide threat to female health with patient outcomes varying widely. The exact correlation between global outcomes of breast cancer and the national socioeconomic status is still undetermined. Mortality-to-incidence ratio (MIR) of breast cancer was calculated with the contemporary age standardized incidence and mortality rates for countries with data available at GLOBOCAN 2012 database. The MIR matched national human development indexes (HDIs) and health system attainments were respectively obtained from Human Development Report and World Health Report. Correlation analysis, regression analysis, and Tukey-Kramer post hoc test were used to explore the effects of HDI and health system attainment on breast cancer MIR. Our results demonstrated that breast cancer MIR was inversely correlated with national HDI (r = -.950; *P* < .001) and health system attainment (r = -.898; *P* < .001). Countries with very high HDI had significantly lower MIRs than those with high, medium and low HDI (*P* < .001). Liner regression model by ordinary least squares also indicated negative effects of both HDI (adjusted R^2^ = .903, standardize β = -.699, *P* < .001) and health system attainment (adjusted R^2^ =. 805, standardized β = -.009; *P* < .001), with greater effects in developing countries identified by quantile regression analysis. It is noteworthy that significant health care disparities exist among countries in accordance with the discrepancy of HDI. Policies should be made in less developed countries, which are more likely to obtain worse outcomes in female breast cancer, that in order to improve their comprehensive economic strength and optimize their health system performance.

## Introduction

Breast cancer is the most frequently diagnosed cancer and the leading cause of cancer death among females, accounting for 25% of all cancer cases and 15% of all cancer deaths worldwide[1]. Based on GLOBOCAN estimates, a total of 1.7 million women were newly diagnosed, and 521,900 died in 2012[1]. However, these global figures hid a wide diversity. The incidence rates were generally higher in more economically developed countries than those in developing ones. In the United States, one in eight women develops breast cancer in her lifetime[2]. The risk of breast cancer in some African countries, such as Mozambique, Malawi, and Rwanda, was below 2%[3]. However, the mortality rates did not correspond proportionally to incidence rates across the world. Mortality-to-incidence ratio (MIR), an approximation of case-fatality rate, is a novel measurement widely used to evaluate whether a country has a higher mortality rate than that might be expected based on its incidence rate[4]. It has also been applied as a valid proxy to estimate the 5-year survival of patients with breast cancer[5].

International variation in the burden of breast cancer reflects distinct differences in risk factors (ie, reproductive, hormonal and lifestyle factors), medical supplies and health infrastructures (ie, the availability of early detection, access to standard treatment), between low- and high-income nations[6, 7]. As we know, the precise correlation between burden of breast cancer and socioeconomic development has not been determined. It is also unclear to what extent disparities in health care services affect the outcomes of female breast cancer. Therefore, our study was aimed to clarify the relationship between the burden of female breast cancer, evaluated by MIR, and the national socioeconomic development assessed according to the global Human Development Index (HDI). Moreover, we also identified the differences in MIR based on the attainment of each country’s health care system.

## Materials and Methods

### Epidemic of global breast cancer

The age-standardized incidence and mortality rates of female breast cancer from 184 countries worldwide in 2012 were obtained from GLOBOCAN 2012 database[8]. Detailed information on the methods used to generate the incidence and mortality estimates for each country is also available on GLOBOCAN website[8]. MIR was calculated as the reported mortality rate for a given country divided by its incidence rate.

### Human development index

The HDIs of 187 Union Nation members in 2012 were obtained from United Nations Development Programme database, according to Human Development Report 2013[9]. HDI is a composite index measuring national well-being in three basic dimensions (four indicators), including long and healthy life (life expectancy at birth), education (mean and expected years of schooling), and decent standard of living (gross national income per capita, GNI). The index ranges from 0 to 1, with higher scores reflecting a greater degree of human development. According to quartiles of HDI distribution in the report, countries were classified into four predefined socioeconomic groups as follows: very high (HDI ≥ .805), high (.805 > HDI ≥ .712), medium (.712 > HDI ≥ .536), and low (HDI < .536).

### Health system attainment

The data of health system attainment from 191 member countries were obtained from the World Health Organization (WHO) World Health Report 2000[10]. The numerical score of health system attainment ranges from 0 to 100, with higher scores reflecting better achievements in the national health system. The index is a weighted average of five different indices: level (25%) and distribution (25%) of health, level (12.5%) and distribution (12.5%) of health care responsiveness, and fairness of financial contribution (25%).

### Statistical analysis

The correlation coefficients of breast cancer MIR with socioeconomic and health care parameters were calculated by Pearson correlation analyses if the hypothesis of normality was confirmed by the Kolmogorov-Smirnov test[11], otherwise Spearman correlation analyses were performed. We performed linear regression analyses, which estimated the average effect of HDI or health system attainment on breast cancer MIR. Quantile regression[12] was further performed to examine if the effects of HDI or health system attainment on MIR differed across quantiles in their conditional distributions. We estimated the model at the .10, .25, .50, .75, and .90 quantiles. The statistical significance of differences in breast cancer MIR among countries in four HDI groups was determined by One-way ANOVA since no heterogeneity of variances was identified[13]. The breast cancer MIR in very high HDI countries was compared with that in high, medium, and low HDI countries using Tukey-Kramer post hoc test[14]. Regression analyses were performed using Stata 12 (Stata Corp, College Station, Tex). Other statistical analyses were conducted with SPSS 20 (IBM-SPSS Inc, Armonk, NY), and results were plotted using GraphPad Prism 6 (GraphPad, San Diego, Calif) and Microsoft Office Excel 2016 (Microsoft, Redmond, WA). *P* values < .05 were considered significant.

## Results

### Breast cancer epidemic and national HDI

**Fig 1:**
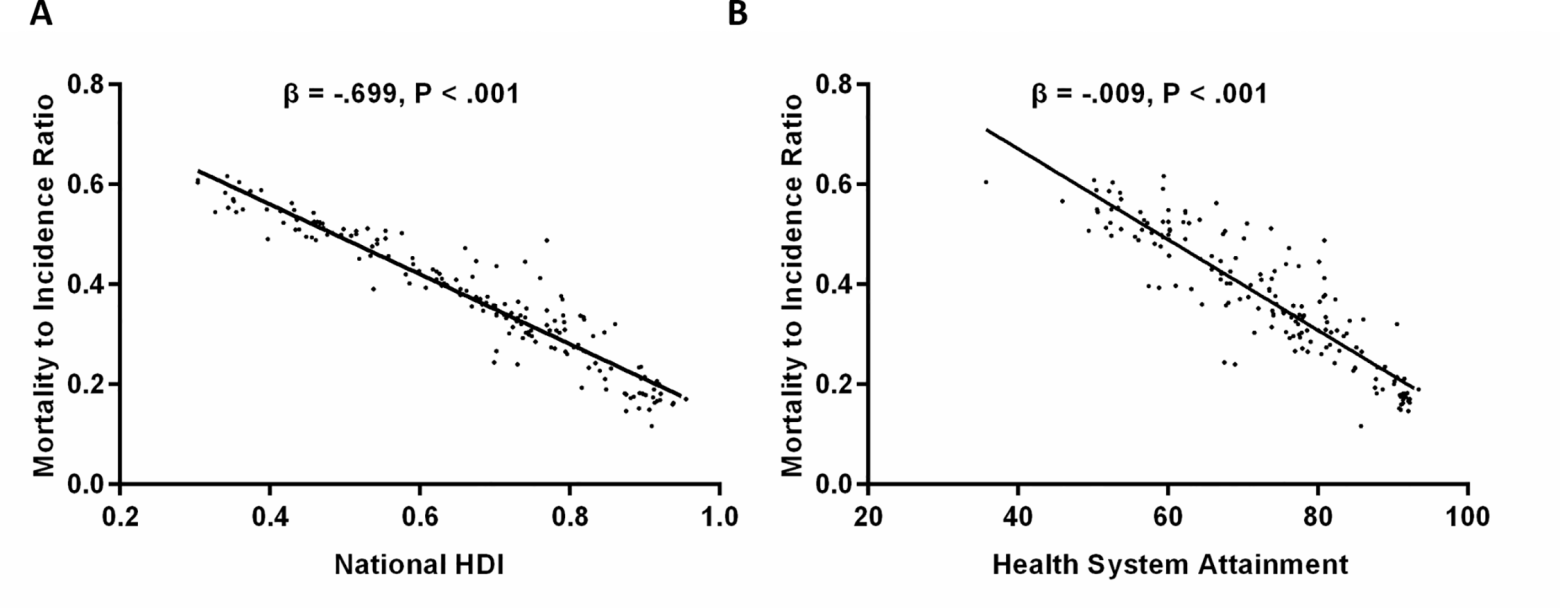
Scatter plots show standardize coefficients of liner regression model between (A) Human Development Index (HDI), (B) health system attainment and breast cancer MIR (mortality-to-incidence ratio).

**Fig 2:**
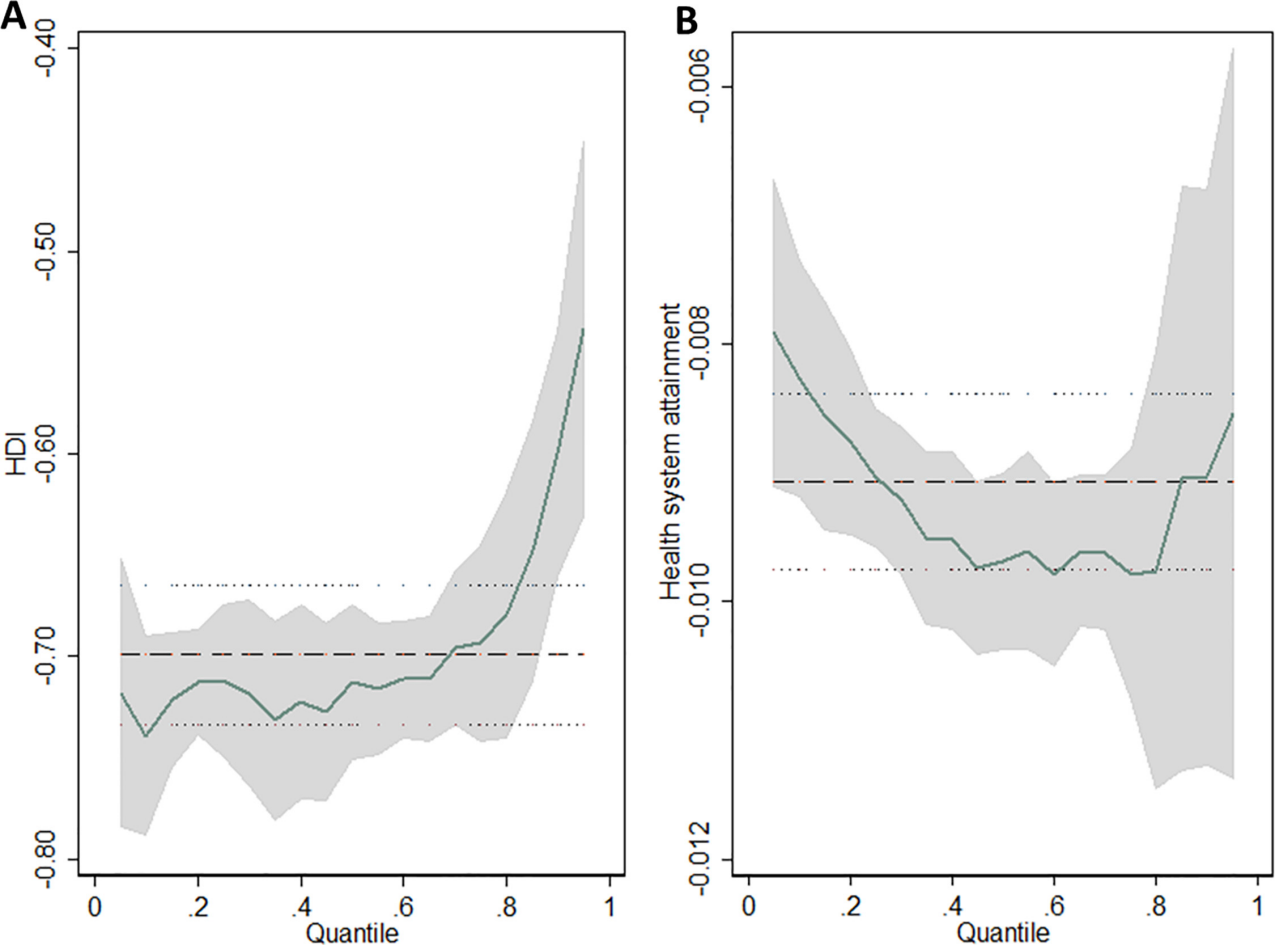
**Variation in the regression coefficients of (A) HDI and (B) health system attainment on breast cancer MIR over the conditional quantiles.** Horizontal lines represent ordinary least squares estimates with 95% confidence intervals.

Data on the MIR of female breast cancer and corresponding national HDI were available in 172 countries. Correlation coefficients revealed that MIR was significantly inversely correlated with national HDI (r = -.950; *P* < .001) and its four indicators (S1 Table). Liner regression model by ordinary least squares (OLS) (Fig 1A) also demonstrated a negative effect of HDI on MIR (adjusted R^2^ = .903, standardize β = -.699, *P* < .001). Quantile regression found that significant negative effects of HDI on breast cancer MRI remained in all quantiles (S2 Table, *P* < .001), and the curve revealed that values of estimated coefficient of HDI on MIR sharply decreased from the 80th percentile (Fig 2A).

**Fig 3:**
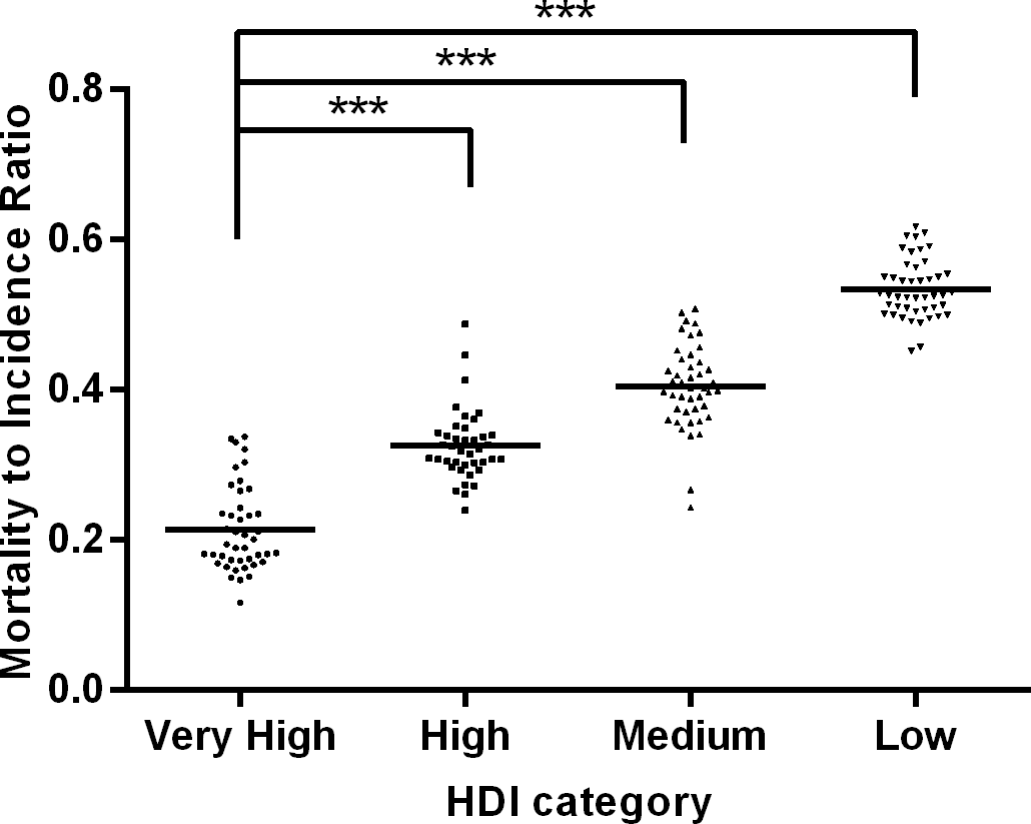
Differences of breast cancer MIR in four socioeconomic development levels. MIR in low, medium and high HDI countries is significantly higher than that in very high HDI countries. ***P< .001. Horizontal lines represent group means.

The 172 countries were placed into four groups according to different national HDIs, including very high (N = 43), high (N = 40), medium (N = 44), and low HDI countries (N = 45). One-way ANOVA indicated that MIR differed significantly among countries in different development levels (*P* < .001). The results of Tukey-Kramer post hoc tests (Fig 3) revealed that MIR in the countries with very high HDI was .214 ± .057 (mean ± standard deviation), which was significantly lower than that in countries with high HDI at .326 ± .047, medium HDI at .405 ± .056, and low HDI at .534 ± .039 (*P* < .001).

**Fig 4:**
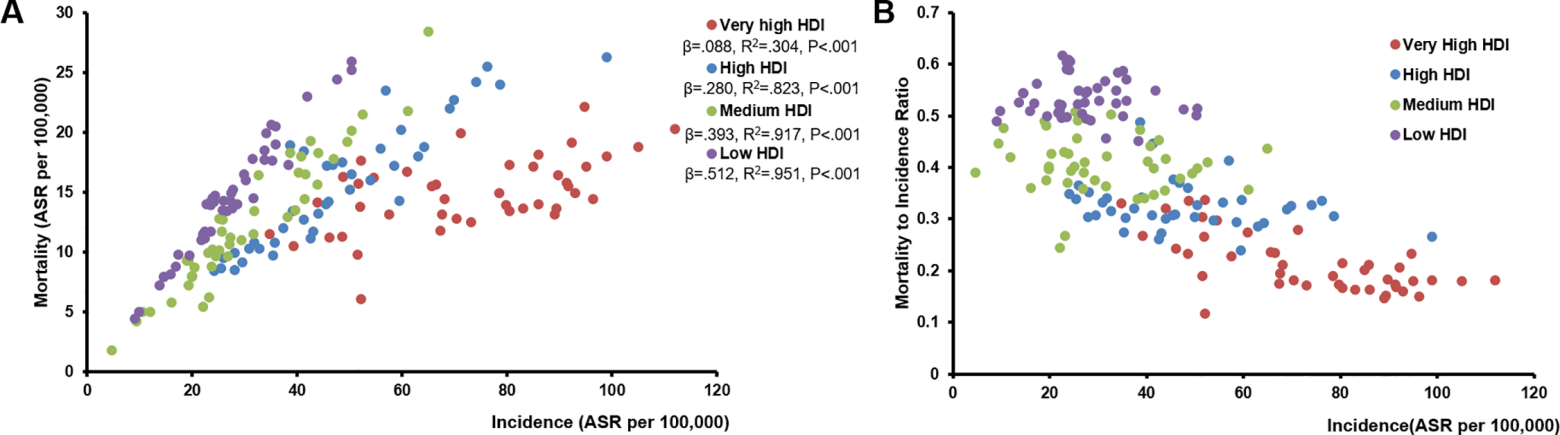
**Scatter plots show the relationship between breast cancer incidence and (A) mortality, (B) MIR of countries in four different HDI categories.** ASR: age-standardised rate; β: standardize coefficient.

Fig 4A showed the relationship of breast cancer incidence and mortality of countries in four different HDI categories. Liner regression model by OLS demonstrated incremental regression coefficients from low to very high HDI countries. And low HDI countries obtained the worst MIR accompanied with low incidence rate (Fig 4B). In those very high HDI countries whose incidence rates were greater than 80 per 100,000, their MIRs reached steady state.

### Breast cancer epidemic and health system attainment

Data on the MIR of female breast cancer and national health system attainment were available in 169 countries. Similarly, health system attainment was inversely correlated with MIR (r = -.898; *P* < .001, S3 Table). Of the five national indices compositing the health system attainment, level and distribution of health, and level of health care responsiveness exhibited strong association with MIR (r = -.897, -.864, and -.845 respectively; *P* < .001). We then evaluated the specific relationship between health system attainment and MIR by both OLS and quantile regression (S2 Table). Liner regression model (Fig 1B) illustrated that health system attainment had a significantly negative effect on breast cancer MIR (adjusted R^2^ = .805, standardized β = -.009; *P* < .001). Furthermore, the quantile regression revealed that health system attainment was remarkably negatively associated with MIR at different quantiles, with a U-shaped curve showing the maximum absolute value of estimated coefficients at .40 to .80 quantiles (Fig 2B).

## Discussion

In current study, we reviewed the global incidence and mortality rates of female breast cancer according to the GLOBOCAN database. MIR, representing case-fatality rate, was significantly inversely correlated with national HDI and its four indicators. Countries with higher HDI levels achieved lower MIRs. Quantile regression curve demonstrated that socioeconomic status had a great negative effect on breast cancer outcomes especially in developing nations with lower HDI and higher case-fatality rates. Health system attainment illustrated a comparable trend with HDI in relation to MIR, indicating that health system performed in higher-quality ensured better consequences in breast cancer patients.

HDI is a composite measure of social and economic development based on the length and quality of life, access to knowledge and living standard[9]. Health system attainment, as a function of achievement in three goals: good health, responsiveness and fairness of financial contribution[10], is used to assess the performance of health systems. It is interesting to note that health system attainment is highly positively correlated with national HDI (S1 Fig, *P* < .001). Countries with more developed socioeconomic status generally adopt more investment in medical health to build better health infrastructure, to provide more educational chances, and also to make greater efforts in the development of screening programmes and novel therapies, all of which may help breast cancer patients achieve better outcomes.

More developed countries account for about one-half of all breast cancer cases in the world but only 38% of all deaths[1]. In Western countries, the mortality rates of breast cancer have become stable since early 1990s [15]. It mainly attributes to early detection through mammographic screening and improvement in cancer treatment[16, 17]. Breast cancer screening has been established in the United Kingdom since 1988[18], which reduced a relative risk of mortality by about 20%[18–20]. By the year 2000, about 70% of women over 40 years in the United States were reported undergoing routine mammography[21]. However, long-term organized mammographic screening programme is too cost-prohibitive to be afforded by those less developed countries. In African countries, for instance, opportunistic or on-demand mammographic screening is only available for those patients with higher social and economic status[3]. Seven in ten of newly diagnosed breast cancer in developed countries are in clinical stage 0 orⅠ, whereas approximately three quarters of women with breast cancer in developing countries are diagnosed in clinical stage Ⅲ or Ⅳ[22].

Over the past three decades, dramatic progress has also been made in breast cancer therapies. In the west world, appropriate treatment was chosen for one patient based on her intrinsic subtype of cancer. However, adjuvant, neoadjuvant, endocrine or targeted therapies in low-resource countries are still gravely underutilized. The age-standardized, 5-year relative survival rate of breast cancer has increased to more than 90% in the United States[23], whereas the rate in Eastern Libya is still only 56%[24].

It is undeniable that deficient early detection programme, delayed disease diagnosis and insufficient treatment are current situation in less developed countries. Meanwhile, for developed countries, there is an ongoing debate about the overdiagnosis and subsequent overtreatment of breast cancer with the prevalence of screening. Further studies are required to make sure whether the certain incidence rate of 80 per 100,000 (Fig 4B) could be used as an upper threshold to argue that overdiagnosis might be taking place. And challenges are still remained in further improving the patients’ long-term survival, especially for women with triple negative, chemo- and/or targeted- therapy resistant or end-stage breast cancer.

HIV/AIDS, tuberculosis, malaria, cholera and many other communicable diseases remain to be major causes of mortality in low-income countries[25]. These diseases of poverty occupied a great majority of health resources and caused the loss of young wealth-producing adults, which in turn resulted in further deterioration of national economy.

Nevertheless, breast cancer is a heterogeneous disease, currently classified into four major intrinsic subtypes―luminal A, luminal B, Her-2/neu and basal cell-like (triple negative)―with distinct prognoses[26,27]. Triple negative breast cancer (TNBC) is an aggressive disease and has poor survival. It was reported that, in developing countries, the proportion of TNBC is much higher, for example 39% in Saudi Arabia, 29% in Malaysia, 23% in China and 17–28% in North Africa, compared with 10–12% in Caucasian women[28]. Luminal A subtype with a favorable clinicopathologic characteristics was more frequent in developed countries. Biological variances in different countries were associated with age of onset, lifestyles and inherited susceptibility for certain patterns of mammary carcinogenesis[28,29]. Besides socioeconomic development and health care services, disparities in the prevalence of specific molecular subtypes may also affect MIRs. In addition, there were great disparities in cancer outcomes across different regions even in the same country. For example, Brazil, a country of continental dimensions with widespread regional and social inequalities, achieved much lower breast cancer mortality rates in south region than those in north and northeast regions, which was in line with their socioeconomic development[30]. In the United States, the MIR also varied from state to state, with lowest ratio in the District of Columbia and highest ratio in the Arkansas. Survival inequalities also persist by ethnicity. African American women had poorest outcomes in the United States compared with other ethnicities[2]. Though our study only focused on the national discrepancies, disparities within a country should be highly valued when a specific country was concerned.

This study is a retrospective analysis about correlations between national HDI, health system attainment and breast cancer MIR. Further studies are still needed to conclude the exact effects of national socioeconomic status and health care services on the outcomes of breast cancer.

## Conclusions

The results of our study revealed that more developed countries, as measured by high HDIs, are inclined to have higher incidence rates of breast cancer in females, but lower MIRs. Low- and middle- income countries with poor health resource settings still carry a heavy burden of breast cancer. Our findings suggest a need to pay more attention to the female breast cancer in less developed countries where medical resource is sparse and communicable diseases remain great threaten to people’s health. These countries should be committed to improve their comprehensive national strengths, promote economic development, and establish better health care systems to realize early detection and sufficient treatment of breast cancer.

## Supporting Information

S1 Fig Scatter plots show standardize coefficients of liner regression model between national HDI and health system attainment. (PDF)

S1 Table Correlation coefficients between HDI, its four indicators, and female breast cancer MIR. (PDF)

S2 Table Comparison of linear and quantile regression results using breast cancer MIR as a dependent variable. (PDF)

S3 Table Correlation coefficients between health system performance and female breast cancer MIR. (PDF)

## References

[1] Torre LA, Bray F, Siegel RL, Ferlay J, Lortet-Tieulent J, Jemal A. Global cancer statistics, 2012. CA: a cancer journal for clinicians. 2015;65(2):87–108. 25651787 10.3322/caac.21262

[2] DeSantis C, Ma J, Bryan L, Jemal A. Breast cancer statistics, 2013. CA: a cancer journal for clinicians. 2014;64(1):52–62. 24114568 10.3322/caac.21203

[3] Kantelhardt EJ, Cubasch H, Hanson C. Taking on breast cancer in East Africa: global challenges in breast cancer. Current opinion in obstetrics & gynecology. 2015;27(1):108–14. 25490377 10.1097/GCO.0000000000000139

[4] Patel AR, Prasad SM, Shih YC, Eggener SE. The association of the human development index with global kidney cancer incidence and mortality. The Journal of urology. 2012;187(6):1978–83. 10.1016/j.juro.2012.01.121 22498216

[5] Vostakolaei FA, Karim-Kos HE, Janssen-Heijnen MLG, Visser O, Verbeek ALM, Kiemeney LALM. The validity of the mortality to incidence ratio as a proxy for site-specific cancer survival. Eur J Public Health. 2011;21(5):573–7. 10.1093/eurpub/ckq120 20813895

[6] Katzke VA, Kaaks R, Kuhn T. Lifestyle and cancer risk. Cancer J. 2015;21(2):104–10. 10.1097/PPO.0000000000000101 25815850

[7] Jemal A, Center MM, DeSantis C, Ward EM. Global patterns of cancer incidence and mortality rates and trends. Cancer epidemiology, biomarkers & prevention: a publication of the American Association for Cancer Research, cosponsored by the American Society of Preventive Oncology. 2010;19(8):1893–907. 10.1158/1055-9965.EPI-10-0437 20647400

[8] GLOBOCAN 2012: Estimated Cancer Incidence, Mortality and Prevalence Worldwide in 2012. Available: http://globocan.iarc.fr Accessed 30 April 2015.

[9] Programme UND. Human Development Report 2013: The Rise of the South: Human Progress in a Diverse World. Available: http://hdr.undp.org/en/content/human-development-report-2013 Accessed 30 April 2015.

[10] Organization TWH. The World Health Report 2000: Health Systems: Improving Performance. World Health Organization 2000 Available: http://www.who.int/whr/2000/en/ Accessed 30 April 2015.

[11] Rosenthal R. An application of the Kolmogorov-Smirnov test for normality with estimated mean and variance. Psychological reports. 1968;22(2):570 5650254 10.2466/pr0.1968.22.2.570

[12] Koenker R, Bassett G. Regression Quantiles. Econometrica. 1978;46(1):33–50.

[13] Pinneau SR, Levine AJ, Schurr BC, Butler DC. Analysis of factor variance: one-way classification. Perceptual and motor skills. 1966;23(3):1209–10. 5972922 10.2466/pms.1966.23.3f.1209

[14] Tukey JW. Comparing individual means in the analysis of variance. Biometrics. 1949;5(2):99–114. 18151955

[15] DeSantis CE, Bray F, Ferlay J, Lortet-Tieulent J, Anderson BO, Jemal A. International Variation in Female Breast Cancer Incidence and Mortality Rates. Cancer Epidemiol Biomarkers Prev. 2015;24(10):1495–506. 10.1158/1055-9965.EPI-15-0535 26359465

[16] Althuis MD, Dozier JM, Anderson WF, Devesa SS, Brinton LA. Global trends in breast cancer incidence and mortality 1973–1997. International journal of epidemiology. 2005;34(2):405–12. 15737977 10.1093/ije/dyh414

[17] Berry DA, Cronin KA, Plevritis SK, Fryback DG, Clarke L, Zelen M, et al Effect of screening and adjuvant therapy on mortality from breast cancer. The New England journal of medicine. 2005;353(17):1784–92. 16251534 10.1056/NEJMoa050518

[18] Independent UK Panel on Breast Cancer Screening. The benefits and harms of breast cancer screening: an independent review. Lancet. 2012;380(9855):1778–86. 10.1016/S0140-6736(12)61611-0 23117178

[19] Pace LE, Keating NL. A systematic assessment of benefits and risks to guide breast cancer screening decisions. Jama. 2014;311(13):1327–35. 10.1001/jama.2014.1398 24691608

[20] Lauby-Secretan B, Scoccianti C, Loomis D, Benbrahim-Tallaa L, Bouvard V, Bianchini F, et al Breast-cancer screening-viewpoint of the IARC Working Group. The New England journal of medicine 2015;372(24):2353–58. 10.1056/NEJMsr1504363 26039523

[21] Swan J, Breen N, Coates RJ, Rimer BK, Lee NC. Progress in cancer screening practices in the United States: results from the 2000 National Health Interview Survey. Cancer. 2003;97(6):1528–40. 12627518 10.1002/cncr.11208

[22] Coughlin SS, Ekwueme DU. Breast cancer as a global health concern. Cancer Epidemiol. 2009;33(5):315–8. 10.1016/j.canep.2009.10.003 19896917

[23] Institute NC. SEER Cancer Statistics Review, 1975–2011.

[24] El Mistiri M, Salati M, Marcheselli L, Attia A, Habil S, Alhomri F, et al Cancer incidence, mortality, and survival in Eastern Libya: updated report from the Benghazi Cancer Registry. Annals of epidemiology. 2015: [Epub ahead of print]. 10.1016/j.annepidem.2015.03.012 25911981

[25] World Health Statistics 2015. World Health Organization 2015 Available: http://www.who.int/gho/publications/world_health_statistics/2015/en/. Accessed 20 May 2015.

[26] Sorlie T, Perou CM, Tibshirani R, Aas T, Geisler S, et al Gene expression patterns of breast carcinomas distinguish tumor subclasses with clinical implications. Proc Natl Acad Sci. 2001;98:10869–74. 11553815 10.1073/pnas.191367098 PMC58566

[27] Sotiriou C, Neo SY, McShane LM, Korn EL, Long PM, et al Breast cancer classification and prognosis based on gene expression profiles from a population-based study. Proc Natl Acad Sci. 2003;100:10393–8. 12917485 10.1073/pnas.1732912100 PMC193572

[28] Corbex M, Bouzbid S, Boffetta P. Features of breast cancer in developing countries, examples from North-Africa. Eur J Cancer. 2014;50:1808–18. 10.1016/j.ejca.2014.03.016 24767469

[29] de Kruijf EM, Bastiaannet E, Ruberta F, de Craen AJM, Kuppen PJK, et al Comparison of frequencies and prognostic effect of molecular subtypes between young and elderly breast cancer patients. Molecular Oncology. 2014;8:1014–25. 10.1016/j.molonc.2014.03.022 24767310 PMC5528523

[30] Gonzaga CM, Freitas-Junior R, Souza MR, Curado MP, Freitas NM. Disparities in female breast cancer mortality rates between urban centers and rural areas of Brazil: ecological time-series study. Breast. 2014;23(2):180–7. 10.1016/j.breast.2014.01.006 24503143

