# Supplementary figures and images for: The Outcome of Breast Cancer Is Associated with National Human Development Index and Health System Attainment
Source: PLoS One. 2016 Jul 8;11(7):e0158951. doi: 10.1371/journal.pone.0158951 (PMC4938431; doi:10.1371/journal.pone.0158951)

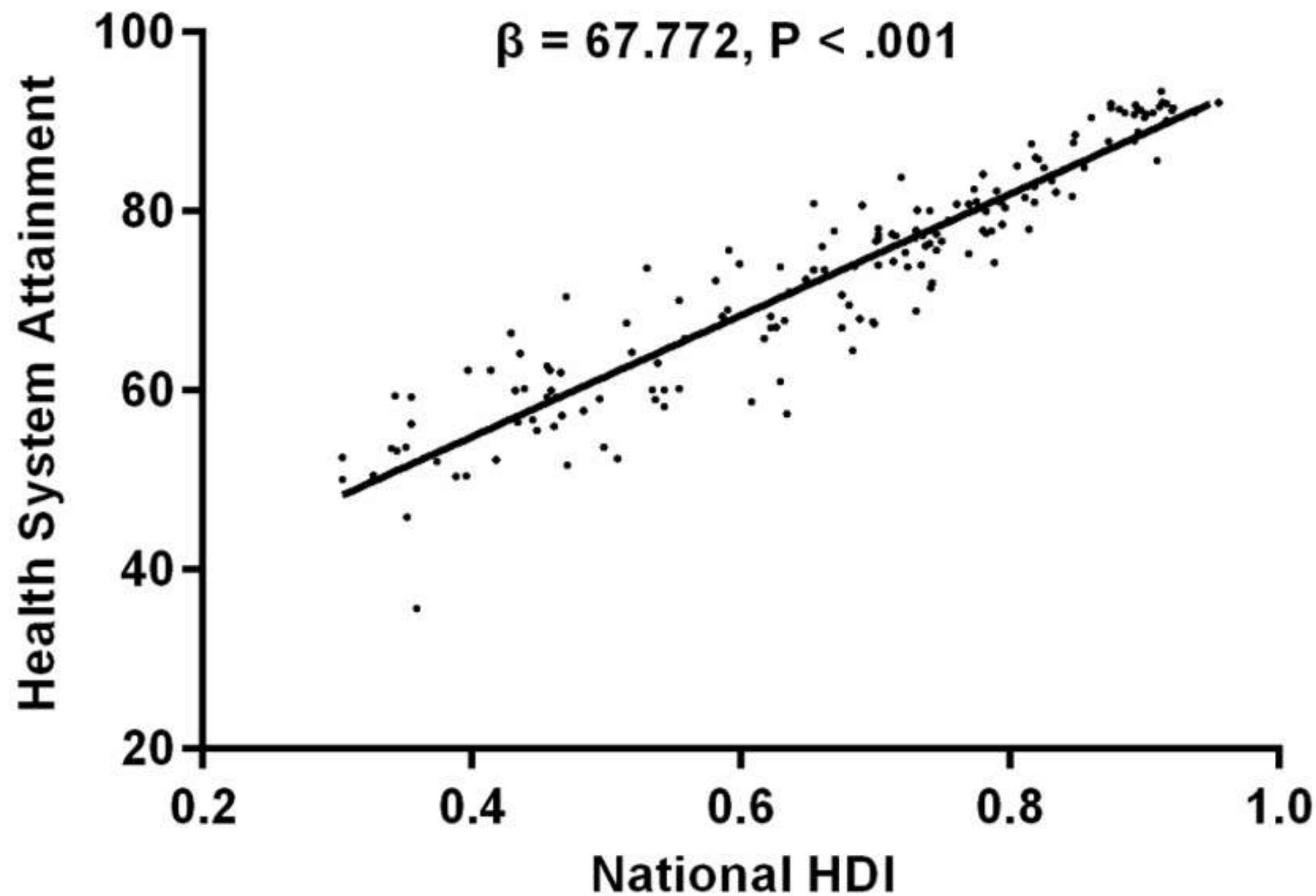

Supplement: S1 Fig — (PDF) [file pone.0158951.s001.pdf]
