# Supplementary material for: The Outcome of Breast Cancer Is Associated with National Human Development Index and Health System Attainment
Source: PLoS One. 2016 Jul 8;11(7):e0158951. doi: 10.1371/journal.pone.0158951 (PMC4938431; doi:10.1371/journal.pone.0158951)
Supplement: S1 Table — (PDF) [file pone.0158951.s002.pdf]

S1 Table. Correlation coefficients between HDI, its four indicators, and female breast cancer MIR.

**Variables related to MIR**	**Correlation coefficients**
**HDI**[a]	-.950***
**Life expectancy at birth, y**[b]	-.895***
**Mean years of schooling**[a]	-.839***
**Expected years of schooling**[a]	-.876***
**Gross national income per capita, $**[b]	-.899***

[a]: Pearson correlation coefficient; [b]: Spearman's rank correlation coefficients after hypothesis of normality was rejected by Kolmogorov-Smirnov test. ****P* < .001.
