# Supplementary material for: The Outcome of Breast Cancer Is Associated with National Human Development Index and Health System Attainment
Source: PLoS One. 2016 Jul 8;11(7):e0158951. doi: 10.1371/journal.pone.0158951 (PMC4938431; doi:10.1371/journal.pone.0158951)
Supplement: S2 Table — (PDF) [file pone.0158951.s003.pdf]

S2 Table. Comparison of linear and quantile regression results using breast cancer MIR as a dependent variable.

Independent variable	OLS regression	Quantile regression				
		.10	.25	.50	.75	.90
**HDI**						
**Regression coefficient**	-.699***	-.739***	-.712***	-.713***	-.693***	-.600***
**Standard error**	.018	.025	.019	.019	.025	.031
**Health system attainment**						
**Regression coefficient**	-.00907***	-.00827***	-.00903***	-.00969***	-.00979***	-.00903***
**Standard error**	.00034	.00046	.00027	.00035	.00050	.00113

OLS: ordinary least squares; ***$P < .001$.
