# Supplementary material for: The Outcome of Breast Cancer Is Associated with National Human Development Index and Health System Attainment
Source: PLoS One. 2016 Jul 8;11(7):e0158951. doi: 10.1371/journal.pone.0158951 (PMC4938431; doi:10.1371/journal.pone.0158951)
Supplement: S3 Table — (PDF) [file pone.0158951.s004.pdf]

S3 Table. Correlation coefficients between health system performance and female breast cancer MIR.

Variables related to MIR	Correlation coefficients
**Health system attainment**[a]	-.898***
**Level of health**[b]	-.897***
**Distribution of health**[b]	-.864***
**Level of health care responsiveness**[b]	-.845***
**Distribution of health care responsiveness**[a]	-.631***
**Fairness of financial contribution**[b]	-.431***

[a]: Pearson correlation coefficient; [b]: Spearman's rank correlation coefficients after hypothesis of normality was rejected by Kolmogorov-Smirnov test. ***P < .001.
